# Characterization With Gene Mutations in Han Chinese Patients With Hypospadias and Function Analysis of a Novel AR Genevariant

**DOI:** 10.3389/fgene.2021.673732

**Published:** 2021-06-30

**Authors:** Lifen Chen, Junqi Wang, Wenli Lu, Yuan Xiao, Jihong Ni, Wei Wang, Xiaoyu Ma, Zhiya Dong

**Affiliations:** Department of Pediatrics, Ruijin Hospital, School of Medicine, Shanghai Jiao Tong University, Shanghai, China

**Keywords:** hypospadias, gene mutations, AR, *SRD5A2*, gene function analysis

## Abstract

It is estimated that around 10–20% of hypospadias are caused by genetic abnormalities worldwide although the spectrum of associated genes does vary across different ethnicities. The prevalence of hypospadias among the Chinese population has been increasing the last couple of decades. However, the pathogenesis underlying the disease and its associated genetic abnormality remains unclear. Here we performed a genetic analysis of 81 children with karyotype 46, XY and the hypospadias phenotype in order to characterize the genetic components that contribute to the development of hypospadias in Chinese patients. 15 candidate genes, including sex determination genes-*SOX9*, *SRY*, *NR0B1* (*DAX1*), *NR5A1* (*SF1*), *DHH*, sex differentiation genes*-AR*, *SRD5A2*, *MAMLD1*, *INSL3*, and hypospadias-associated genes*-FGF8*, *FGF10*, *BMP4*, *BMP7*, *ATF3*, and *MID1* were screened by using next generation sequencing. A total of 18 patients were found to have mutations identified by PCR and sequencing, including 11 cases of *SRD5A2* genes, 6 cases of *AR* genes, and 1 case of *MID1* gene, respectively. One novel missense mutation p.I817N was discovered in *AR* gene. Further molecular analysis found that subcellular localization of the AR_I__81__7N_ was the same as that of wild type AR_WT_ in the absence or presence of hormone. But it led to 50% reduction in AR-induced transcriptional activity in the presence of either the synthetic androgen R1881 or the natural ligand dihydrotestosterone. Our results indicate that *SRD5A2* and *AR* genes are two top candidate genes associated with 46, XY hypospadias in Chinese patients. Further epidemiological and genetic analysis are still needed to further clarify the pathogenesis of hypospadias in Han Chinese patients.

## Introduction

Hypospadias is one of the most common congenital malformations of the penis (van der Horst and de Wall, 2017; Donaire and Mendez, 2018). It is characterized by a failure of urethral groove closure resulting in an opening on the ventral surface of the penis. The prevalence of hypospadias varies according to ethnical and geographical differences. The reported prevalence in Western countries ranges from 2 to 43 out of 10,000 live births (Springer et al., 2016). In China, the prevalence was 0.7–4.5 from 1996 to 2008, and 5.8 per 10,000 live births in 2010 (Jin et al., 2010), but has increased to 9.3 per 10,000 live births as of 2012 (Li et al., 2012).

Although the exact etiology of hypospadias is unknown, it is agreed upon that environment, endocrine hormones, and genetic factors all play a collective role in the pathogenesis (Carmichael et al., 2012). Environmental factors such as assisted reproductive technology, maternal hypertension during pregnancy, thyroid disease, higher maternal age at delivery, low birth weight, preterm birth, and primiparity have been proposed. Most diagnosed cases of hypospadias are sporadic, but family cluster genetic phenomena do occur (Donaire and Mendez, 2018).

Genetic disruption of male external genital development and urethral growth contribute to 20% of the etiology of hypospadias (Domenice et al., 2000; Gangaher et al., 2016). There are three key pathways in the development of male external genitalia: (a) androgen independent, (b) androgen dependent, and (c) pathways dependent on endocrine and environmental factors (Manson and Carr, 2003). The steroid 5α-reductase 2 (SRD5A2) and androgen receptor (AR) genes are two androgen dependent genes that were widely evaluated in hypospadias. Among the Chinese population, SRD5A2 and AR dysfunction have been discovered in some patients with hypospadias. The AR is an intracellular transcription factor and plays a crucial role in male sex differentiation. In both sporadic and familial hypospadias, AR gene mutations have been discovered. Hypospadias is one of the major phenotypes of partial androgen insensitivity syndrome (PAIS) (Joodi et al., 2019). To date, over 1,000 mutations have been reported in the AR gene, and it is usually included as one of the top genes in the screening panel test for patients with hypospadias. Moreover, not only are their mutations reported in the coding region of AR, but also SNPs located in the promoter region of AR gene were involved in the development and severity of hypospadias (Geller et al., 2014; Pineyro-Ruiz et al., 2020) however, the functional importance of these mutations remains less known. AR gene mutations also lead to several pathological situations such as androgen insensitivity syndrome (AIS), spinal and bulbar muscular atrophy (SBMA), and prostate cancer (Shukla et al., 2016).

To explore the spectrum of genetic abnormality in Chinese hypospadias patients, we performed next generation sequencing (NGS) on 81 patients with hypospadias targeting 15 candidate genes that could potentially cause hypospadias including (1), sex determination genes: sex determining region of Y chromosome (*SRY*), SRY-related high mobility group box gene 9 (*SOX9*), nuclear receptor subfamily 0 group B member 1 (*NR0B1*), nuclear receptor subfamily 5 group A member 1 (*NR5A1*), desert hedgehog (*DHH*) (2), sex differentiation genes: *SRAD5, AR*, mastermind-like domain-containing protein 1 gene (*MAMLD1*), Leydig cell insulin-like 3 (*INSL3*) (3), and hypospadias genes reported by other studies: bone morphogenetic protein gene 4 and 7 (*BMP4* and *7*), fibroblast growth factor 8 and 10 (*FGF8* and *10*), activating transcription factor 3 (*ATF3*), and midline 1 (*MID1*). Multiple mutations were found in these patients, but not in 50 healthy controls. One of 4 identified *AR* missense mutations was *de novo* and functional impairment of AR from this mutation was further explored *in vitro*.

## Materials and Methods

### Patients

Patients with external genital malformations who were followed in the Department of Pediatrics at Ruijin Hospital, Shanghai Jiao Tong University School of Medicine from January 2004 to December 2020 were screened initially. 81 cases with karyotype 46, XY and phenotype of hypospadias were identified and included in this study. Those patients who had syndrome related external genital malformations were excluded. Informed written consent was obtained from all patients’ parents or their guardians. In addition, 50 boys without external genital malformation were selected as controls for genetic analysis. The study was approved by the Institutional Review Board (Ethics Committee of Shanghai Jiao Tong University School of Medicine).

### Clinical Assessment

Clinical assessment of these patients included the child’s birth history, family history, maternal history (exogenous sex hormone exposure or intake history during pregnancy), living environment and possible history of exposure to environmental pollutants. Age, gender, body weight, gonadal developmental status, degree of external genital malformation according to the external masculinization score (EMS), and type of hypospadias were recorded. The measurement of penis length and reference value of Chinese men were performed according to the published study by Fu Chao et al. in 2010 (Fu Chao and Xuliang, 2010). The diagnosis of cryptorchidism in these patients was made according to the study performed by Bao et al. (Bao and Zhang, 2012). External Masculinization Score (EMS, range 0–12) (Ahmed et al., 2000) was used to assess the degree of virilization of the external genitalia. The normal male score is 12 and a smaller score indicates a lower virilization degree. An EMS < 7 is considered ambiguous.

### Gonadotropin-Releasing Hormone (GnRH) Agonist Testing and Human Chorionic Gonadotropin (hCG) Stimulation Test

GnRH agonist testing was performed in some patients using intravenous administration of a standard dose of GnRH (2 ug/kg Gonadorelin acetate, maximum dose 100 ug). Blood samples were collected at time points 0, 30, 60 and 90 min after stimulation. Normal test was defined as luteinizing hormone (LH) increases about 3–6 times within 30–45 min, and follicle stimulating hormone (FSH) increases by 20–50% after GnRH injection. Serum LH and FSH were measured by immunoradiometric assay (Abbott, Chicago IL). The hCG stimulation test which was used to evaluate the function of testicular synthetic androgens was performed in some patients with a daily injection of hCG (2,000 U) for 3 days. Serum testosterone (T) and dihydrotestosterone (DHT) were measured using a radioimmunoassay kit (Diagnostic Systems Laboratories, Webster, Tex., United States).

### DNA Extraction, PCR and Sequencing

Genomic DNA was extracted from the peripheral whole blood using the FlexiGene DNA Kit (Qiagen GmbH, Hilden, Germany), according to the manufacturer’s instructions. 15 genes (FGF8, FGF10, BMP4, BMP7, NR5A1, MAMLD1, SRY, SOX-9, NR0B1, DHH, ATF3, INSL3, MID1, SRD5A2, and AR) according to literature search were selected as candidates for polymerase chain reaction (PCR) and sequencing. Primers targeting all exons of these genes were designed using online tool Primer3^1^. PCRs were performed in a reaction volume of 50 μl containing 20 ng template DNA. A 5 μl aliquot of each PCR was loaded on a 2% agarose gel and visualized by ethidium bromide (Sigma-Aldrich, Beijing, China) staining to confirm the presence of an appropriately sized product. The PCR products were purified using a gel extraction kit (QIAGEN, Mississauga, Ont., Canada) and sequenced in both sense and antisense directions on an Illumina-miseq sequencer (Illumina, United States). DNA fragments were 250–400 bp and the coverage of samples was 80x–3,000x. Positive and negative controls were added to the samples. Sequences generated from all patients were compared with the published reference sequences from the National Center for Biotechnology Information^2^. The mutation was annotated according to HGVS nomenclature^3^. For identified mutation from NGS, purified PCR products were further sequenced and verified by Sanger sequencing on an ABI 700 sequencer (Applied Biosystems PerkinElmer, Foster City, Calif., United States). The ACMG guidelines (Amendola et al., 2016) were used to classify the mutations into five classifications: pathogenic (P), likely pathogenic (LP), variants of uncertain significance (VUS), likely benign (LB), or benign (B).

### Site-Directed Mutagenesis and Construction of AR Expression Vectors

The wild type AR expression plasmid (AR_WT_) (SC114220) was bought from Origene (Rockville, MD). It served as a template to construct the mutant p.I817N (AR_I__81__7N_) using the QuikChange II site-directed mutagenesis kit (Agilent Genomics, Santa Clara, CA, United States). The primers were ARt2450aF: ccatccactggattaatgctgaagagtagcagtgct andARt2450aR: agcactgctactcttcagcattaatccagtggatgg. Positive clones were selected and sequenced to confirm the site-directed mutation (Du et al., 2009).

### Immunofluorescence Staining

The cultured cells were fixed and then followed by overnight incubation with primary antibody anti-AR (Invitrogen). Sections were washed and then incubated with Cy2-anti-mouse and Cy5 anti-rabbit (Jackson Immuno Research, West Grove, PA). Nuclear stating was performed using 4′,6-diamidino-2-phenylindole (DAPI; Molecular Probes, Eugene, OR). Confocal microscopy was performed using a Leica SP2AOBS system (Leica, Wetzlar, Germany) in the Light Microscopy Core Center at the Shanghai Jiao Tong University.

### Western Blot

Cells harvested from cultures were homogenized in cell lysis buffer (Cell Signaling Technology, Danvers, MA). Protein concentration determination and immunoblotting were performed as previously described. Briefly, twenty micrograms of protein were separated by SDS-PAGE, transferred to polyvinylidene difluoride membranes and blotted with primary antibodies and secondary antibodies (Cell Signaling Technology, Danvers, MA). Quantification of the image was performed by scanning densitometry and using NIH Image J 1.54 software (National Institutes of Health, Bethesda, MD).

### The Transcription Activity Assay

The transactivation activity of the p.I817N mutated AR and wild-type AR were compared in a reporter gene assay as previously described (Du et al., 2009). In brief, Chinese hamster ovary cells (CHO) were transfected with a total of 100 ng DNA per well, consisting of the expression vector (AR_WT_ or AR_I__81__7N_) and the Cignal reporter plasmid (QIAGEN, Germantown, MD) using transfection reagent Lipofectarmine 2000 (Invitrogen, Grand Island, NY, United States). Cells were incubated overnight with dihydrotestosterone (DHT; 0.01–30 nM), methyltrienolone (R1881; 0.001–100 nM), and hydroxyflutamide (OHF; 1–5,000 nM) each in triplicate. For OHF treatment, 0.1 nM of R1881 was added 30 min before the addition of OHF to determine the competition binding. Then cells were lysed and luciferase activity assayed using Victor^3^ plate reader (Perkinelmer, Waltham, MA).

### Statistical Analysis

Statistical analyses were performed using an unpaired, two-tailed *t*-test for comparing between AR_WT_ and AR_I__87__1N_. The one-way ANOVA was used for comparing cellular responses after R1881 treatment. The clinical data description was not involved in the statistics. Calculations were performed using Graphpad Prism 9 software. Significance was determined using a threshold of *P* = 0.05. All values were reported as mean ± standard error of the mean (SEM) for three independent experiments.

## Results

### Clinical Findings of Patients With Hypospadias

All 81 patients diagnosed with hypospadias exhibited other features of external genital malformations at variable degrees as listed in Table 1. The age of patients ranged from 3 months to 22.2 years and the average age is 6.1 ± 5.1 years old. According to the location of the urethral meatus, hypospadias was classified into four types. Type I was glandular or coronal, type II was shaft (distal, mid and proximal), type III was scrotal and type IV was perineal. The majority of patients (52/81) were found to have severe types (III and IV). 18 (22.2%) patients were raised as female after birth due to obscure external genitalia, short penis, and poor scrotal development like the labia majora. Except for external genital malformation, some children were also accompanied by other abnormalities, such as being small for their gestational age, inguinal hernia, atrial septal defect and breast development during adolescence. A positive family history was found in 2 children, one of whom had a history of hypospadias in both his father and younger brother, and the other had a cousin diagnosed with hypospadias.

**TABLE 1:** Clinical characteristics of 81 patients diagnosed with hypospadias.

Type of hypospadias	I	II	III	IV	Total
Number of cases (%)	9 (11.1%)	14 (17.3%)	31 (38.3%)	27 (33.3%)	81 (100%)
EMS (mean)	7.8	6.9	6.0	5.5	6.2
**Other features of ambiguous genitalia**					
Micropenis	5 (55.5%)	7 (50%)	24 (77.4%)	13 (48.1%)	49 (60.5%)
Microtesticle	2 (22.2%)	1 (7.1%)	12 (38.7%)	13 (48.1%)	28 (34.6%)
Cryptorchidism	3 (33.3%)	2 (14.3%)	16 (51.6%)	14 (51.9%)	35 (43.2%)
Clubbed penis	0	3 (21.4%)	4 (12.9%)	4 (14.8%)	11 (13.6%)
Poor scrotum	2 (22.2%)	3 (21.4%)	13 (41.9%)	20 (74.1%)	38 (46.9%)
Hydrocele	0	0	3 (9.7%)	3 (11.1%)	6 (7.4%)
Testicular microlithiasis	1 (11.1%)	1 (7.1%)	2 (6.5%)	1 (3.7%)	5 (6.2%)
Epididymal head cyst	0	1 (7.1%)	0	2 (7.4%)	3 (3.7%)
**Other associated clinical features**					
Inguinal hernia	0	1 (7.1%)	2 (6.5%)	3 (11.1%)	6 (7.4%)
SGA	1 (11.1%)	2 (14.3%)	2 (6.5%)	4 (14.8%)	8 (9.8%)
ASD	0	0	0	5 (18.5%)	5 (6.2%)
Adolescent mammoplasia	0	4 (28.6%)	1 (3.2%)	2 (7.4%)	7 (8.6%)
Positive family history	0	1 (7.1%)	1 (3.2%)	0	2 (24.5%)

**EMS, external masculinization score; SGA, small-for-gestational age; ASD, atrial septal defect.**

Forty-two cases received GnRH stimulation test as a follow up. Seven patients showed high gonadotrophin dysplasia and the remaining cases had normal response. Fifty-two patients underwent the hCG challenge test, and only five patients had a poor response (the T concentration increased less than 3 times of the baseline value after stimulation). Other patients demonstrated a normal testicular response to hCG.

### Identification and Characterization of Nucleotide Substitutions

G banding method was used for karyotype analysis and all children have 46, XY chromosomes. 15 candidate genes were selected for the initial screening using Illumina-miseq sequencing according to previous literatures. All mutations detected in the initial screening by NGS were confirmed by traditional Sanger sequencing. A total of 18 patients were found to have mutations, including 11 cases of SRD5A2 genes, 6 cases of AR genes, and 1 case of MID1 gene. According to ACMG guidelines, these mutations were assessed as pathogenic (P) or likely pathogenic (LP) (Table 2). The presence of these mutations was further tested in 50 normal controls to rule out polymorphisms.

**TABLE 2:** Genotype hypospadias patients with candidate gene abnormities.

Patient ID	Gene	cDNA	Protein	Exon	Rs number	Genotype	Origin	ACMG
1	*SRD5A2*	G680 > A	R227Q	E4	rs9332964	CompoundHet	Mother	P/PS3 + PM1 + PM2 + PM3 + PP4
		G737 > A	R246Q	E5	rs9332967		Father	P/PS3 + PM1 + PM2 + PM3 + PP4
2	*SRD5A2*	G680 > A	R227Q	E4	rs9332964	Hom	Mother/Father	P/PS3 + PM1 + PM2 + PM3 + PP4
3	*SRD5A2*	G607 > A	G203S	E4	rs9332961	CompoundHet	Mother	P/PS3 + PM1 + PM2 + PM3 + PP4
		G680 > A	R227Q	E4	rs9332964		Father	P/PS3 + PM1 + PM2 + PM3 + PP4
4	*SRD5A2*	G607 > A	G203S	E4	rs9332961	Hom	Mother/Father	P/PS3 + PM1 + PM2 + PM3 + PP4
5	*SRD5A2*	C408 > A	Y136Ter	E2	/	CompoundHet	*De novo*	P/PVS1 + PM2 + PM6 + PP3
		G680 > A	R227Q	E4	rs9332964		Mother	P/PS3 + PM1 + PM2 + PM3 + PP4
6	*SRD5A2*	A578 > G	N193S	E5	rs763296857	CompoundHet	Mother	P/PS3 + PM1 + PM2 + PM3 + PP4
		G737 > A	R246Q	E5	rs9332967		Father	P/PS3 + PM1 + PM2 + PM3 + PP4
7	*SRD5A2*	G737 > A	R246Q	E5	rs9332967	Hom	Mother/Father	P/PS3 + PM1 + PM2 + PM3 + PP4
8	*SRD5A2*	C59 > T	L20P	E1	rs761824859	CompoundHet	Mother	LP/PM1 + PM2 + PP3 + PP4 + PP5
		G680 > A	R227Q	E4	rs9332964		Father	P/PS3 + PM1 + PM2 + PM3 + PP4
9	*SRD5A2*	C59 > T	L20P	E1	rs761824859	Hom	Mother/Father	LP/PM1 + PM2 + PP3 + PP4 + PP5
10	*SRD5A2*	A578 > G	N193S	E5	rs763296857	CompoundHet	Father	P/PS3 + PM1 + PM2 + PM3 + PP4
		656delT	F219fs*60	E5	rs61748127		Mother	P/PVS1 + PS3 + PM2 + PM3
11	SRD5A2	G607 > A	G203S	E4	rs9332961	CompoundHet	Father	P/PS3 + PM1 + PM2 + PM3 + PP4
		656delT	F219fs*60	E5	rs61748127		Mother	P/PVS1 + PS3 + PM2 + PM3
12	*AR*	C2338 > T	R780W	E6	/	Hem	Mother	P/PS3 + PM1 + PM2 + PM3 + PP4
13	*AR*	T2450 > A	I817N	E7	/	Hem	*De novo*	LP/PS2 + PM1 + PP3 + PP4
14	*AR*	G2567 > A	R856H	E7	rs9332971	Hem	Mother	P/PS3 + PM1 + PM2 + PM3 + PP4
15	*AR*	C2612 > T	A871V	E8	rs143040492	Hem	Mother	P/PS3 + PM1 + PM2 + PP4 + PP5
16	*AR*	Exon 5-8 gross deletion, chX 67716101-67724136			/	Hem	Mother	P/PVS1 + PM2 + PP3 + PP4
17	*AR*	Exon 2 gross deletion, chX 67643255-67643407			/	Hem	Mother	P/PVS1 + PM2 + PP3 + PP4
18	*MID1*	C2000 > T	P667L	E10	rs147106995	Hom	Mother/Father	LP/PM1 + PM2 + PP3 + PP4

**P, pathogenic; LP, likely pathogenic.**

### Patients With *SRD5A2* Gene Mutations

There were 11 cases identified with SRD5A2 mutations. The age of diagnosis ranged from 4-month to 22-years old. The clinical characteristics are listed in Table 3. Among them, 8 patients were seen before puberty (0.3–5.6 years old) and 3 cases were raised as females. There was one case of type II hypospadias that was not accompanied by any other external genital malformations, 7 cases of type III and 3 cases of type IV. The average EMS of these 9 patients was 4.9. The GnRH test was basically normal, but four children underwent hCG stimulation and were found to have increased T/DHT values. Genetic analysis showed 7 heterozygous mutations (p.R227Q/p.R246Q, p.G203S/p.R227Q, p.Y136Ter/p.R227Q, p.N193S/p.R246P, p.L20P/R227, p.N193S/p.F219Sfs^∗^60, and p.G203S/F219fs^∗^60) and 4 homozygous mutations (p.R227Q, p.G203S, p.R246P, p.L20P). 10 of them were reported in the literature. Only p.Y136Ter was a newly identified mutation, where cytosine at position 408 of exon 2 of SRD5A2 gene mutated to adenine causing the stop codon TAA and terminating translation. According to ACMG guidelines, this mutation was assessed as pathogenic (P/PVS1 + PM2 + PM6 + PP3).

**TABLE 3:** The clinical features of 11 patients carrying SRD5A2 mutations.

No.	Age at diagnosis	Gender upon diagnosis	Type of hypospadias	Complications			EMS	LH/FSH (mIU/ml)	T/DHT (HCG stimulation before/after)
				Microtesticle	Micropenis	Cryptorchidism			
1	0.6	Male	III	Y	Y	Y	2	0.48/3.94	10/10.5
2	2.7	Male	III	Y	Y	N	6	0.5/1.55	–
3	1.9	Female	III	N	Y	Y	1	2.9/7.76	–
4	12.6	Male	IV	N	Y	N	3	0.1/4.26	81.9/–
5	22.2	Male	II	N	N	N	10	2.6/1.8	–
6	2.7	Male	III	N	Y	N	6	4.21/7.71	–
7	0.9	Male	IV	N	Y	N	6	3.3/2.6	–
8	0.3	Female	IV	Y	Y	N	3	2.19/4.06	25.1/30.5
9	10.9	Female	III	Y	Y	Y	5	0.4/0.35	39.7/64.4
10	5.6	Male	III	N	Y	N	6	< 0.07/0.15	8.3/–
11	5.4	Male	III	N	Y	N	6	0.08/0.54	10.9/10.4

**EMS, external masculinization score; LH, Luteinizing Hormone; FSH, Follicle Stimulating Hormone; T, testosterone; DHT, Dihydrotestosterone.**

### Patients With *AR* Gene Mutations

There were 6 cases with an identified AR gene mutation, the clinical phenotype characteristics are shown in Table 4. Three children were younger (1.6, 0.3, and 5.9 years old) at the diagnosis and the other three had reached adolescence (14.6, 14.3, and 14.2 years old) before seeking medical care. 4 children were born with a female gender due to poorly developed scrotum-like labia. After comprehensive consideration of chromosomal factors, genitalization of the external genitalia, and psychological acceptance of children and parents, 2 patients were changed to male support, and 2 remained in female support. Three cases of hypospadias were type IV, one cases was type III, and the other two were type II. All abnormalities of AR gene were hemizygous mutations (p.R780W, p.I817N, p.R856H, and p.A871V) and included two gross deletions (Exon 5-8 gross deletion, chX 67716101-67724136, and exon 2 gross deletion chX 67643255-67643407). p.I817N was the newly discovered missense mutation and not found in the 50 normal controls. This site was located at the beginning of exon 7 of the AR gene, and the 245th thymine was mutated to adenine, resulting in the amino acid at position 817 being changed from isoleucine to asparagine. According to ACMG guidelines, this mutation was assessed as likely pathogenic (LP/PS2 + PM1 + PP3 + PP4). Exon 5-8 gross deletion (chX 67716101-67724136) removed 8035bp including the totality of exon 5-8 and exon 2 gross deletion (chX 67643255-67643407) removed 152 bp containing total exon 2 of AR gene, both were assessed as pathogenic (P/PVS1 + PM2 + PP3 + PP4).

**TABLE 4:** The clinical features of 6 patients carrying AR mutations.

No.	Age at diagnosis	Gender upon diagnosis	Type of hypospadias	Complications			EMS	LH/FSH (mIU/ml)	T/DHT (HCG stimulation before/after)
				Microtesticle	Micropenis	Cryptorchidism			
12	1.6	Female	IV	N	N	Y	5	2.26/2.39	–
13	14.6	Female	IV	N	Y	N	3	27.7/6.7	–
14	14.3	Female	II	N	Y	N	6	18.5/8.6	19.6/21.46
15	0.3	Male	II	N	Y	N	4	1.2/0.83	5.83/–
16	5.9	Male	III	N	N	Y	7	9.95/21.93	2.2/3.6
17	14.2	Female	IV	Y	Y	Y	1	0.82/4.63	–

**EMS, external masculinization score; LH, Luteinizing Hormone; FSH, Follicle Stimulating Hormone; T, testosterone; DHT, Dihydrotestosterone.**

### Patients With MID1 Mutations

One patient carried a homozygous mutation in the *MID1* gene, p.P667L, which was located in exon 10 of the MID1 gene and was the last amino acid encoded by the gene. MID1 gene mutation is associated with X-linked Opitz G/BBB syndrome (Fontanella et al., 2008; Preiksaitiene et al., 2015; Maia et al., 2017). The patient with this mutation which was identified in our study only had hypospadias without other symptoms or abnormalities. But according to ACMG guidelines, this mutation was assessed as likely pathogenic (LP/PM1 + PM2 + PP3 + PP4).

### Expression and Sub-Cellular Distribution of AR_I__81__7N_

**FIGURE 1:**
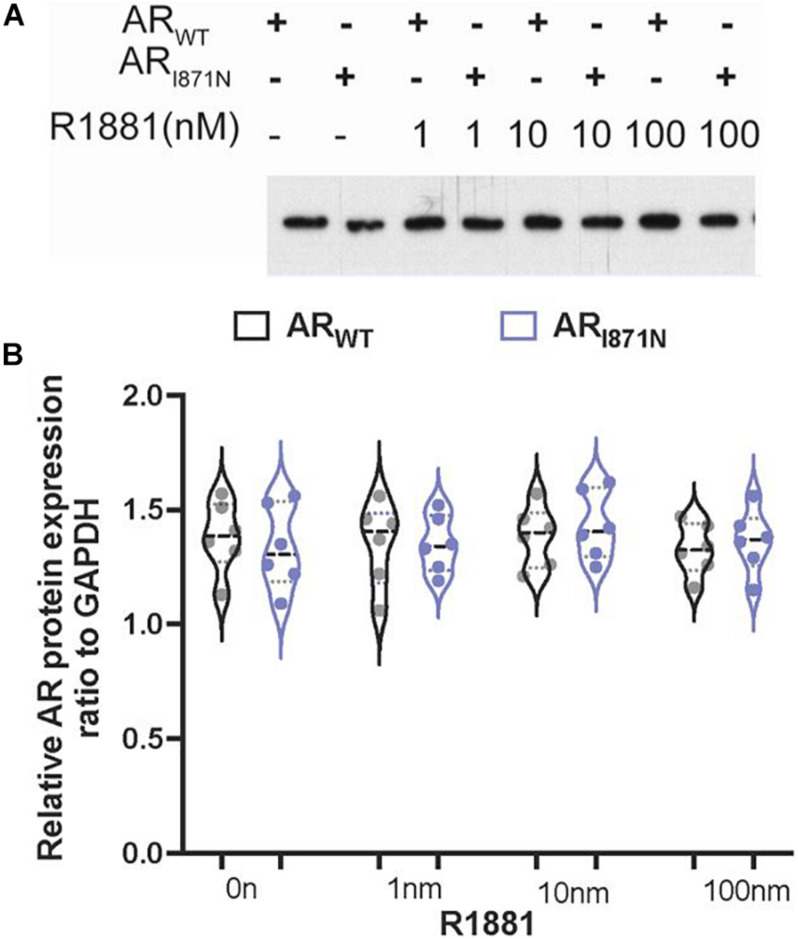
The protein expression of AR_WT_ and AR_I87__1N_ in transfected cells. **(A)** The expression levels of AR_WT_ and AR_I87__1N_ were analyzed in cells 24 h post-transfection in the presence of R1881 (1, 10, and 100 nM) or vehicle alone (0.1% DMSO). An amount of 10 ug lysate was subjected to SDS-PAGE and immunoblotting with AR monoclonal antibody. No significant differences of expression were found between AR_WT_ and AR_I87__1N_. **(B)** Quantitation the protein expression levels of AR.

To determine whether the expression of the AR or the protein stability was affected by this newly identified mutation, site directed mutation was performed using wild type *AR* gene. An immunoblot was performed after incubation of CHO cell line transiently expressing AR_WT_ or AR_I__81__7N_, respectively, with different concentrations of R1881 or vehicles for 24 h. The protein expression level of AR_I__81__7N_ appeared to be similar at every R1881 concentration (Figures 1A,B). These data rule out any severe change in protein stability in the absence or presence of ligand.

**FIGURE 2:**
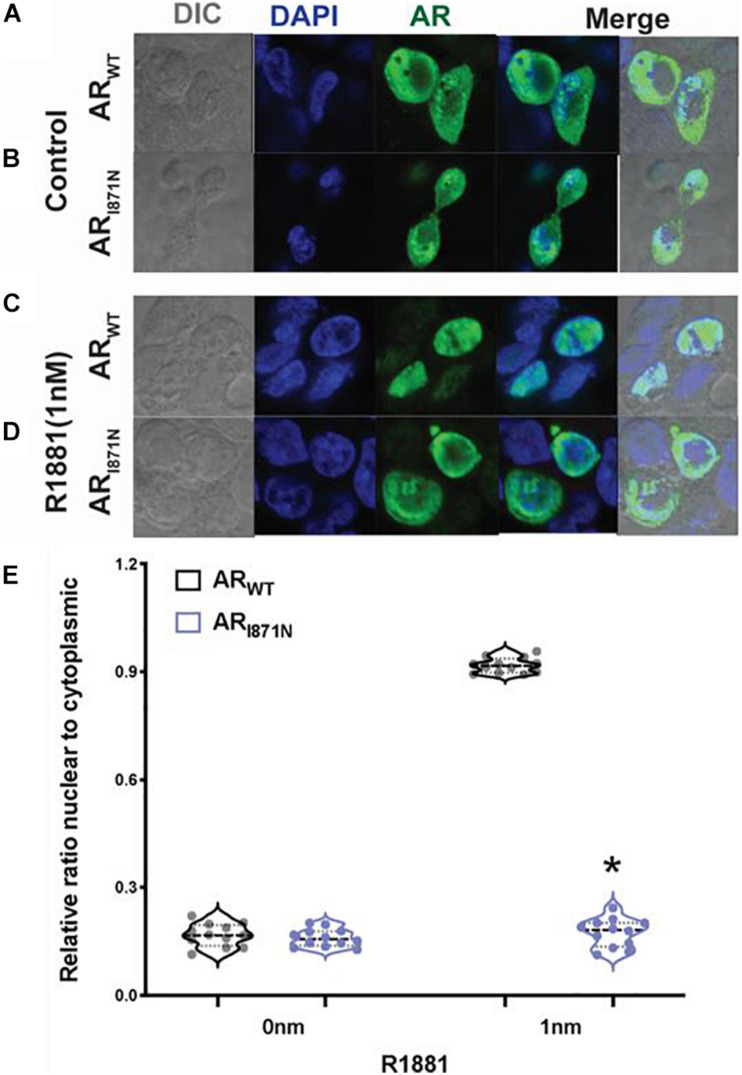
Sub-cellular localization of AR_WT_ and AR_I87__1N_. Confocal microscope images of CHO cells transfected with 1 ug of AR_WT_ or AR_I87__1N_ in the absence of ligand **(A,B)** or in the presence of 1 nM R1881 **(C,D)**. The bars represent 10 μm. **(E)** Quantitation of the ratio of nuclear/cytoplasmic expression of AR. *P < 0.05 compared with wild type.

To investigate whether the p.I817N mutation of *AR* gene could influence AR expression, we further analyzed the subcellular localization of this mutant in CHO cell line. Cells were transfected with AR_WT_ or AR_I__87__1N_ plasmid DNAs, and the localization of AR protein was examined using confocal microscopy. Both AR_WT_ and AR_I__87__1N_ were predominantly located in the cytoplasm in the absence of hormone (Figures 2A,B,E). In the presence of 1nM R1881 both AR_WT_ and AR_I__87__1N_ were translocated in a similar way to the nucleus and displayed a typical punctuate nuclear distribution (Figures 2C–E).

### Transcriptional Activity of AR_I__81__7N_

**FIGURE 3:**
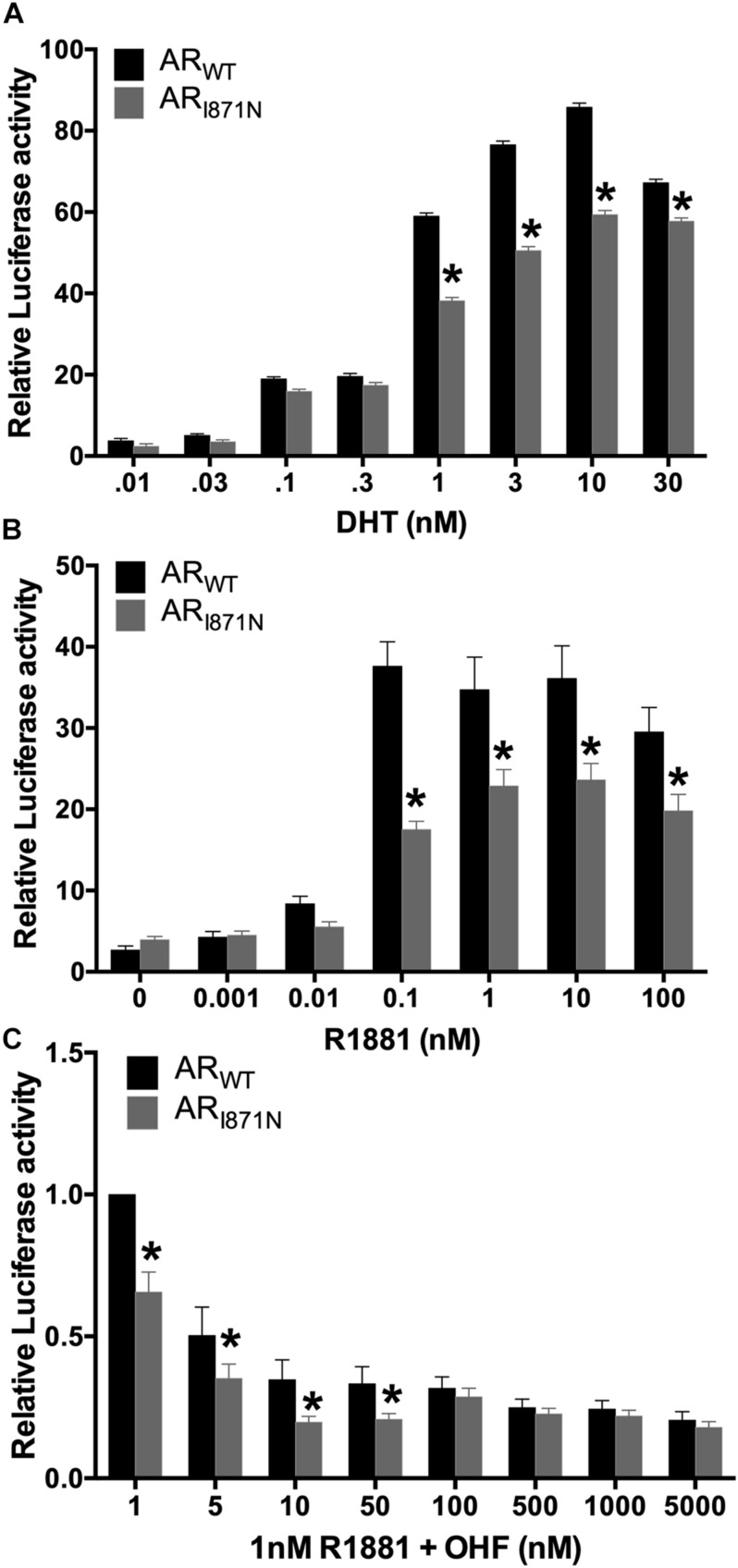
Decreased transactivation activity of AR_I87__1N_ mutation in reporter gene assay. CHO cells transfected with either AR_WT_ or AR_I87__1N_, were exposed to DHT **(A)** or R1881 **(B)** at the different concentrations. In a competitive binding assay, where R1181 competed with the anti-androgen OHF for AR binding sites, the AR_I87__1N_ mutation showed slightly weaker binding than the wild type **(C)**. Lower activity of AR_I87__1N_ was observed when it was compared to the wild-type receptor. The experiments were conducted in triplicate. *P < 0.05 compared with wild type.

The AR_I__87__1N_ mutation is located in the ligand binding domain (LBD) of the AR, which may play a role in coactivator mediated activation of the transcription. Therefore, functional studies were performed to address whether the AR_I__87__1N_ affects the transcription activation potential of AR. The AR_I__87__1N_ or AR_WT_ expression vector was co-transfected with Cignal reporter in luciferase assay. In dose-response curve, the mutated AR showed reduced ability to induce AR-mediated activation of a reporter gene, compared to wild-type AR assessed by the natural ligand DHT or R188. p.I817N caused a 20∼46% reduction in activity at doses between 1 and 10 nM DHT or 0.1–100 nM R1881 (Figures 3A,B). In addition, this was further illustrated by using competitive binding assay with R1881. The mutated AR showed weaker competitive binding than the wild type (Figure 3C). These results suggest that p.I817N mutation leads to a decreased sensitivity of the androgen response.

## Discussion

The prevalence of hypospadias is increasing in China (Li et al., 2012). To explore the potential contribution of genetic components in these patients, we screened 81 children with karyotype of 46, XY who had hypospadias using NGS of 15 target genes. 18 out of 81 patients were found to have mutations. The identified genetic abnormalities of the novel missense mutation *AR* p.I817N were analyzed. It was also noted that 11 mutations in *SRD5A2* gene, 6 mutations in *AR* gene, and one variant in *MID1* gene in this study were also correlated to abnormal male genital development and hypospadias. The genetic abnormality is 22.2% (18/81) which is higher than reported in the literature which has previously suggested that 10–20% of hypospadias are caused by genetic abnormalities (Carmichael et al., 2012; Kalfa et al., 2019). It supports the hypothesis that hypospadias is a multifactorial disease and is a consequence of combination of both environmental and genetic abnormalities.

Male phenotypic development can be divided into two stages: sex determination and differentiation (Domenice et al., 2000). Genetic mutations in genital differentiation and external genital development can cause hypospadias. Based on previous reports, we have selected 15 genes which are potentially involved in the development of hypospadias. During male sexual development, expression of *SRY* induces a series of gene activation, including its direct downstream gene *SOX9*. They mainly act in the early stage of gender differentiation, and their mutations lead to the development of disorders of sexual development (Harley et al., 2003). *NR5A1* is an important transcription factor in male sexual development and steroid synthesis. INSL3 regulates the expression of steroidogenic factor-1, which is an important regulator in the development of gonads and adrenal glands (Sadeghian et al., 2005). *NR0B1* regulates the early stage of testicular differentiation, and its mutation most often causes adrenal hypoplasia congenita and gonadal dysplasia. *DHH* belongs to Hedgehog secretory signaling molecule family. *DHH* gene mutation can cause 46, XY partial gonadal dysplasia. In this study, 81 patients with hypospadias were screened and we did not identify any genetic mutations in these six sex determination genes.

Genetic research on hypospadias has focused on identification of causal mutations too. Ethnic variation of genetic contribution of hypospadias has been described in the literature (Beleza-Meireles et al., 2007; van der Zanden et al., 2012). In Chinese patients, mutations or SNPs in *ATF3*, *FGF8*, *FGF10*, *BMP4*, *BMP7*, and *MAMLD1* have been reported. *ATF3* has a regulatory effect on cell growth (Liu et al., 2005; Kalfa et al., 2008). Co-expression and interaction among *FGF8*, *FGF10*, *BMP4*, and *BMP7* participate in the early stage of male genital development (Morgan et al., 2003; Suzuki et al., 2003). *MAMLD1* is a candidate to explore in patients with unexplained 46, XY DSD, as it has been shown to be expressed in fetal Leydig cells around the critical period for sexual development (Chen et al., 2010). The transient knockdown of *MAMLD1* mRNA expression results in significantly reduced testosterone production in mouse Leydig tumor cells and an *NR5A1* target site was found within the *MAMLD1* gene (Ogata et al., 2009). We found *MAMLD1* p.N662S variant in two patients with isolated hypospadias, which were also identified by other hypospadias mutation studies (Kalfa et al., 2012). Based on ACMG guidelines and reference to the ClinVar database, and HGMD databases, the variant was classified into benign (B).

MID1, as a transcriptional regulatory protein, is involved in the development of the mid-segment structure during embryonic development. Opitz syndrome (OS) is a multiple congenital anomaly disorder that shows a wide spectrum of severity and a highly variable expressivity (OMIM 300000) (Opitz, 1987). In male OS patients, mutations have been found to be scattered throughout the entire length of the *MID1* gene suggesting a loss of function mechanism as the basis of this developmental phenotype (Cox et al., 2000). Hypospadias of all grades was found more commonly in males with MID1 mutations than in those without (So et al., 2005). The patient in our study carrying *MID1* variant p. P667L only has isolated hypospadias, this loci was assessed as likely pathogenic. Recently MID1 was found to up-regulate AR protein levels in several prostate cancer cell lines and its expression was negatively regulated by androgen signaling (Kohler et al., 2014). Like the AR gene, MID1 is expressed in the genital tubercle (GT) in human embryos (Pinson et al., 2004). However, If MID1 loss-of-function mutations as seen in OS led to decreased AR protein levels in the GT, this may help explain the urogenital anomalies seen in OS (Winter et al., 2016).

The *SRD5A2* gene encoded 5α-reductase is mainly expressed in male genital and prostate tissues, whose defects cause 46, XY DSD due to defects in testosterone metabolism. In this study, 13.6% (11/81) hypospadias is caused by SRAD5 mutation, which takes 61.1% (11/18) genetic etiology of hypospadias. A *de novo* mutation p.Y136Ter leads to a stop codon TAA and terminates the translation of SRD5A2 gene. This loci is assessed as pathogenic. The patient carries compound heterozygote p.Y136Ter/p.R227Q and exhibits type II hypospadias, with no external genital malformations except hypospadias. The EMS score was 10 and hCG stimulation tests showed non-response.

So far, more than 1000 cases of mutations with *AR* defects have been reported in the literature, presenting with a wide variety of phenotypic outcomes. The phenotypes of 46, XY individuals with *AR* gene mutations are categorized as complete androgen insensitivity syndrome (CAIS) or PAIS, respectively, and the more severe forms belong to disorders of sex determination. They range from a complete male-to-female sex reversal in CAIS patients to variable grades of ambiguous genitalia in PAIS patients. Milder forms of PAIS also include male phenotypes with gynecomastia, decreased fertility, or isolated hypospadias. PAIS could be a consequence of partial defect of AR structure or function caused by defects in *AR* gene. In this study, 7.4% (6/81) hypospadias was caused by *AR* mutation, which accounted for 33.3% (6/18) genetic abnormality of hypospadias.

The six mutations of the AR gene were found in 6 patients, of which 3 were previous identified mutation sites (p.R780W, p.R856H, p.A871V). p.I817N was a novel missense mutation. Other two patients have gross deletions of AR gene and both were not reported. All 4 missense mutations were located within the highly conserved carboxy-terminal LBD of the *AR* gene. The LBD domain consists of 12 contiguous alpha helices, most of which are hydrophobic amino acids that form a hydrophobic pocket which in turn binds the androgen ligand through its hydrophobic interaction. Functional impairment after mutation in this region weakens the binding of androgen to the AR protein in the target cell resulting in a decrease in the activity of androgen. It has been reported that p.A871V mutation was associated with a decrease in the binding affinity between AR and androgen by approximately 56%. p.R856H mutation led to changes in AR tertiary structure, resulting in decreased thermal stability and mutual N/C interactions. Large deletions in AR gene have been reported in patients with CAIS (Li et al., 2011; Doehnert et al., 2015), but in our data, two patients were not complete female phenotype (EMS7 and 1).

The p.I817N mutation identified in our study changed thymine to adenine at position 2450 in the *AR* gene. It is located in the LBD region of AR and encodes the first amino acid of exon 7, which is involved in the splicing of amino acid chains during transcription and translation. Conservative analysis of 10 mammalian proteins suggested that this region is highly conserved. The concentration of T in the laboratory test was 9.37 ng/ml, and it was 11.95 ng/ml after hCG challenge. It was significantly higher than the serum T level in boys of the same age. This also supports its molecular diagnosis. *In vitro* functional assay was further utilized and revealed that this mutation led to impaired AR transcriptional activity (20∼46%), decreased sensitivity for androgen ligand, and was responsible to hypospadias observed in the patient.

To exclude the correlation between the identified mutations and clinical manifestations other than hypospadias, we performed Fisher’s exact test which was used to compare the incidences of inguinal hernia, SGA, ASD, and puberty breast development in children with and without candidate genes mutations. There were no statistical differences (*P* > 0.05). This study used a candidate gene strategy, there may be unknown gene variation. We cannot rule out the association of these complications with other gene variations.

In conclusion, genetic involvement of hypospadias was explored in 81 Chinese children using NGS. One *de novo* missense mutation loci was identified in AR and further *in vivo* and *in vitro* functional studies provided the molecular evidence that p.I817N amino acid change could cause significant reduction in AR transcriptional function which led to hypospadias. Our results suggest that genetic mutation is one of many factors contributing to the development of hypospadias in Chinese patients as the mutation was not detected in 78% of our patients, but still our data expand the spectrum of mutations in the SRD5A2 gene and AR gene in patients with hypospadias. Recent studies propose that *in utero* exposure to estrogens found in pesticides used in fruits and vegetables as well as in plastic linings can have an anti-androgenic activity (Donaire and Mendez, 2018). Epidemiological and genetic analysis are still needed to further clarify the pathogenesis of hypospadias in Chinese patients.

## Data Availability Statement

The data presented in the study are deposited in the China National GeneBank (CNGB) Nucleotide Sequence Archive (CNSA: https://db.cngb.org/cnsa) repository, accession number CNP0001939. The data are also available on request from the corresponding author, ZD and XM to dzy831@126.com and 179788825@qq.com.

## Ethics Statement

The studies involving human participants were reviewed and approved by the Ethics Committee of Shanghai Jiao Tong University School of Medicine. Written informed consent to participate in this study was provided by the participants’ legal guardian/next of kin.

## Author Contributions

LC was responsible for the writing and editing of this manuscript. LC and JW conducted the experiments. WL, YX, JN, and WW elaborated clinical data. XM and ZD were responsible for the editing of this manuscript. All authors contributed to the article and approved the submitted version.

## Conflict of Interest

The authors declare that the research was conducted in the absence of any commercial or financial relationships that could be construed as a potential conflict of interest.

## Footnotes

1 http://primer3.ut.ee/

2 http://www.ncbi.nlm.nih.gov/

3 http://www.HGVS.org/varnomen
